# Supplementary material for: Synthesis and Characterization of Amine and Aldehyde-Containing Copolymers for Enzymatic Crosslinking of Gelatine
Source: Int J Mol Sci. 2024 Mar 1;25(5):2897. doi: 10.3390/ijms25052897 (PMC10931651; doi:10.3390/ijms25052897)
Supplement: Supplementary file 1 [file ijms-25-02897-s001.zip › ijms-2891780-supplementary.pdf]

International Journal of *Molecular Sciences*

*Supplementary Materials*

# Synthesis and Characterization of Amine and Aldehyde-Containing Copolymers for Enzymatic Crosslinking of Gelatine

**Silvana Alfei *, Federica Pintaudi and Guendalina Zuccari**

Department of Pharmacy (DIFAR), University of Genoa, Viale Cembrano, 4, 16148 Genoa, Italy
* Correspondence: alfei@difar.unige.it

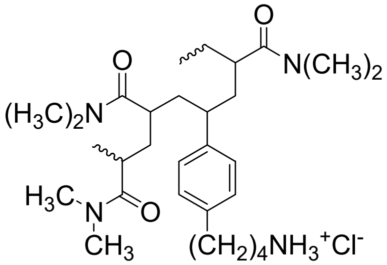


**Figure S1.** Linear LO substrate copolymer (P1c) previously prepared by us of interest for this new study.

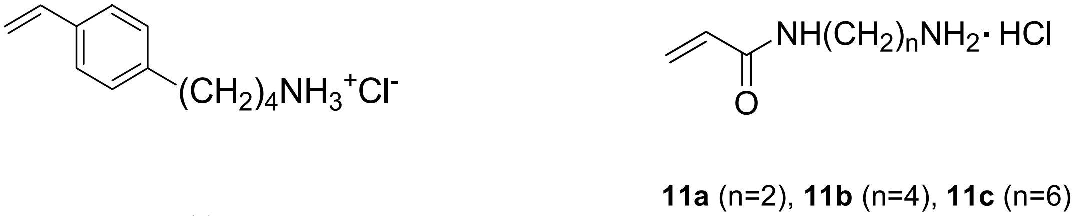


**Figure S2.** Chemical structure of monomer **5** (a); chemical structure of amidoamine monomers **11a-c** (b).

**Table S1.** Copolymerization of **5** with DMAA at 60 °C.

| 5 (mg, mmol) | DMAA (g, mmol) | $M_5$ | AIBN (mg, %) | DMF (mL) | Time (h) | Isolation solvent | Copolymer (g, %) |
|---|---|---|---|---|---|---|---|
| 50.4, 0.238 | 2.5, 25.23 | 0.009 | 25.9, 1.02 | 11.0 | 3 h 30′ | DIPE | CP5/DMAA-0.9 * 2.07, 80.9 |
| 75.4, 0.356 | 2.31, 23.29 | 0.015 | 24.7, 1.04 | 11.0 | 3 h 30′ | DIPE | CP5/DMAA-1.5 * 1.74, 72.9 |
| 155.4, 0.734 | 1.38, 13.95 | 0.052 | 15.8, 1.03 | 7.1 | 24 | $Et_2O$ | CP5/DMAA-5.2 * 0.53, 34.4 |
| 151.2, 0.714 | 0.637, 6.43 | 0.111 | 7.9, 1.00 | 3.6 | 24 | $Et_2O$ | CP5/DMAA-11.1 * 0.504, 63.9 |
| 101.4, 0.479 | 0.421, 4.25 | 0.113 | 5.99, 1.15 | 3.0 | 24 | $Et_2O$ | CP5/DMAA-11.3 * 0.117, 22.3 |
| 698.0, 3.295 | 0.766, 7.73 | 0.426 | 15.3, 1.05 | 7.5 | 7 | $Et_2O$ | CP5/DMAA-42.6 * 0.678, 46.3 |
| 953.0, 4.50 | 1.04, 10.48 | 0.429 | 21.3, 1.07 | 9.5 | 24 | $Et_2O$ | CP5/DMAA-42.9 * 0.860, 81.4 |
| 811.0, 3.83 | 0.88, 8.88 | 0.431 | 17, 1.01 | 8.0 | 21 | $Et_2O$ | CP5/DMAA-43.1 * 0.599, 35.4 |

DMAA = *N, N′*-dimethylacrylamide; $M_5$ = molar fraction of **5** in the feed; AIBN= 2,2'-azo*bis*-(2-methyl-proprionitrile); DIPE= di-isopropyl ether; * the copolymer code number indicates the percentage of **5** in the polymerization feed.

**Table S2.** Results from characterization analyses performed on CP5/DMAA-42.9.

| Entry | µequivNH$_2$/g * | Mn | Z $_{AVE}$ (nm) | ζ-p(mV) | PDI | mmolNH$_2$/g ** | β | β $_{AVE}$ |
|---|---|---|---|---|---|---|---|---|
| CP5/DMAA-42.9 | 894±12 | 5100 | 334 ± 27 | +57.6 ± 1.7 | 1.012 ± 0.007 | 12.5 ± 0.03 | 0.667 | 0.2305 ± 0.1354 |

* Volumetric titration; ** potentiometric titrations; PDI = polydispersion index. AVE = average; β = buffer capacity (dV $_{(HCl)}$/d (pH); β $_{AVE}$ = average buffer capacity. Defined as the volume of HCl necessary to cause a variation of pH equal to one unit in the pH range 4.5–7.5.

**Table S3.** Solubility of DMAA homopolymer (HP-DMAA) and of prepared copolymers using **5**, DMAA and AA.

| Solvent | CP5/DMMA-1.5 * | CP5/DMAA-42.9 * | CP5/DMMA/AA | HP-DMAA |
|---|---|---|---|---|
| Petrol | - | - | - | - |
| $Et_2O$ | - | - | - | - |
| Toluene | Swells on heating | Swells on cooling | - | Partially soluble |
| THF | Partially soluble | Partially soluble | - | Soluble on heating |
| Dioxane | Swells on cooling | Swells on cooling | - | Soluble on heating |
| Acetone | Partially soluble | - | - | + |
| $CHCl_3$ | + | - | Partially soluble | + |
| DCM | + | - | Swells on cooling | + |
| MeOH | + | + | + | + |
| DMF | + | + | + | + |
| DMSO | + | + | + | Soluble on heating |
| $H_2O$ | + | + | + | + |

Petrol = petroleum ether 40–60 °C; DMAA = *N, N′*-dimethylacrylamide; AA = acrylic acid; THF = tetrahydrofuran; DCM = dichloromethane; DMF = *N, N*-dimethylformamide; DMSO = dimethyl sulfoxide; * the copolymer code number indicates the percentage of **5** in the feed.

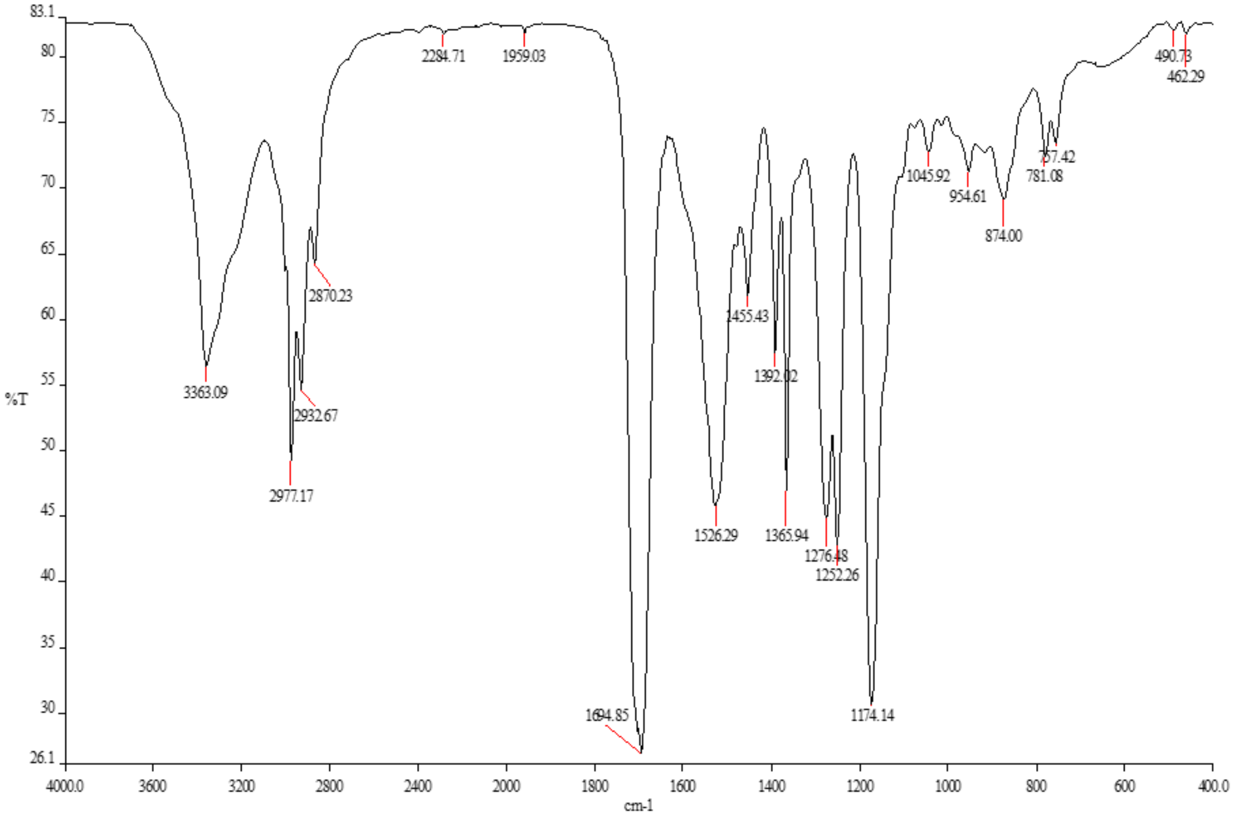


**Figure S3.** FTIR (film) spectrum of **6a**.

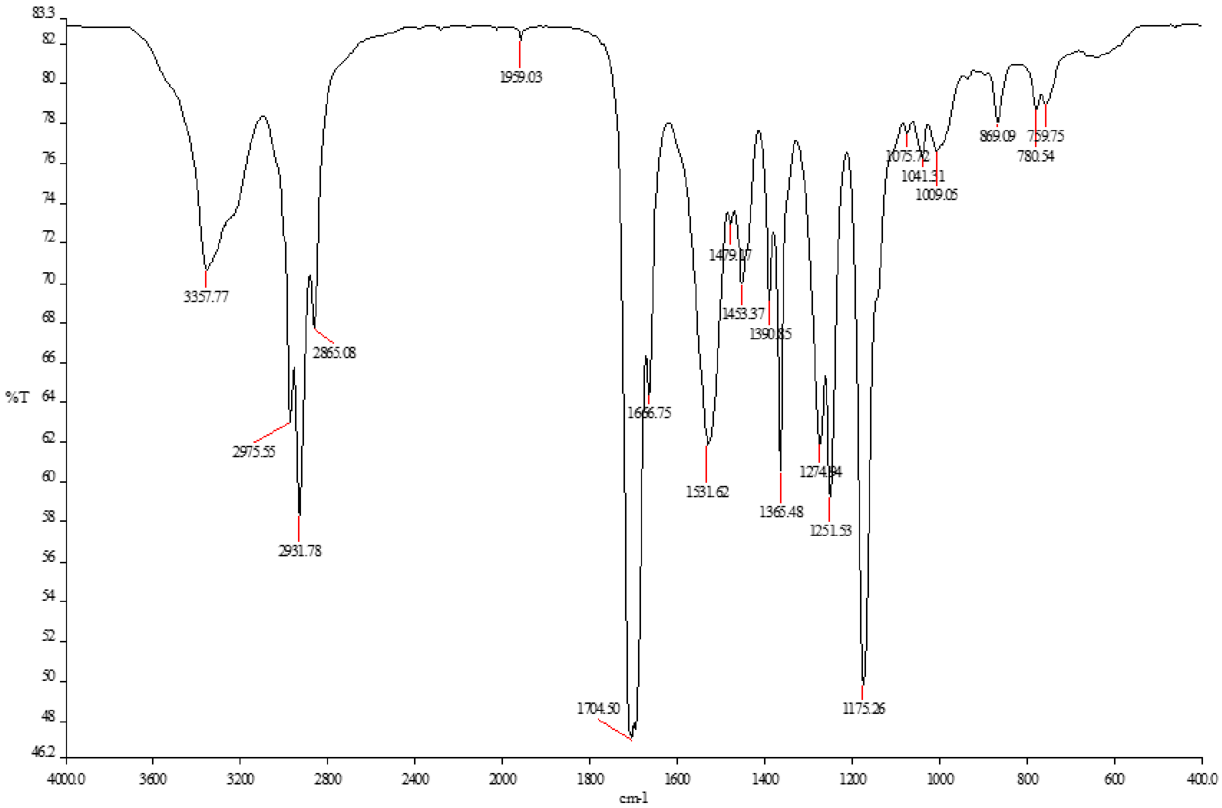


**Figure S4.** FTIR (film) spectrum of **6b**.

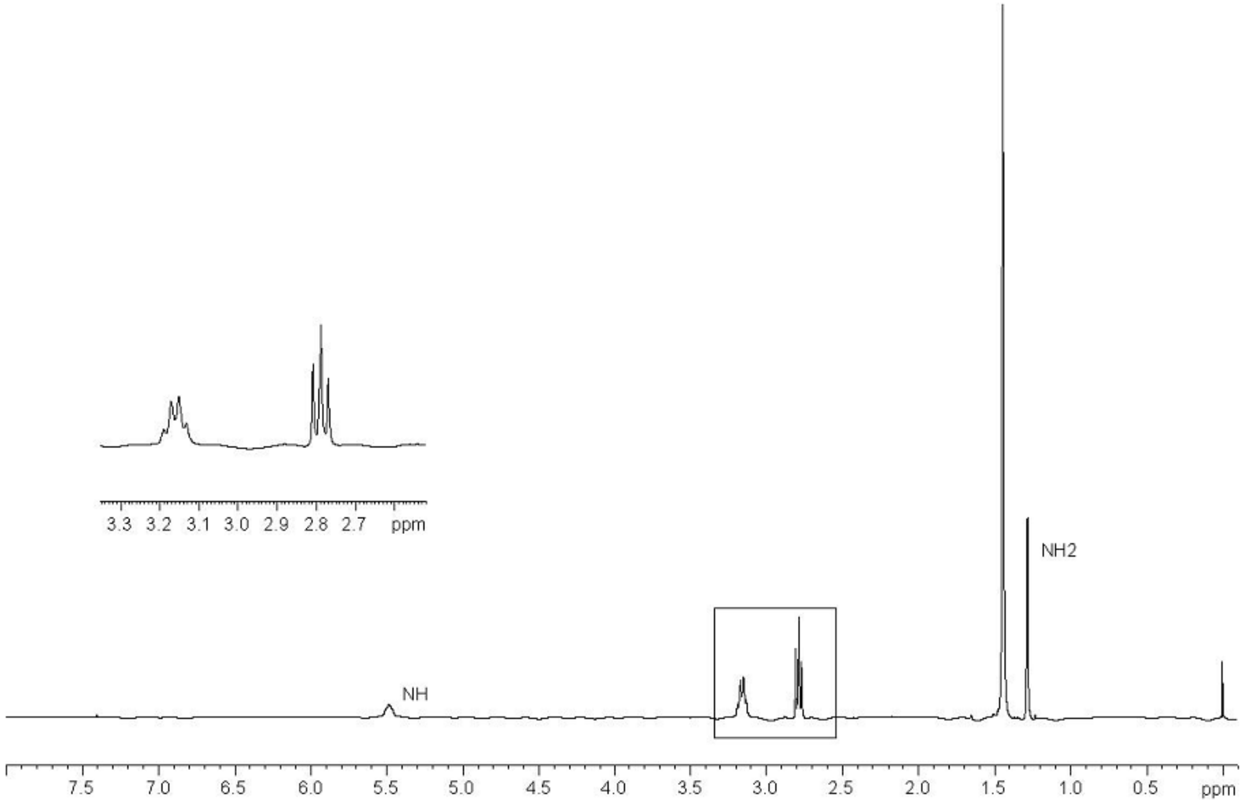


**Figure S5.** $^{1}H$ NMR ($CDCl_3$, 300 MHz) spectrum of **6a**.

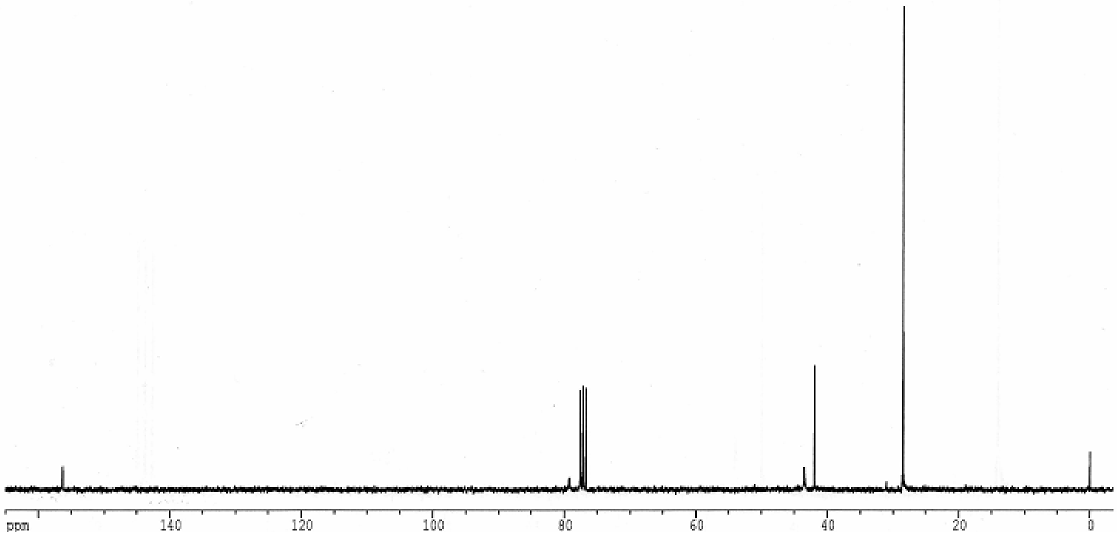


**Figure S6.** $^{13}C$ NMR ($CDCl_3$, 75.5 MHz) spectrum of **6a**.

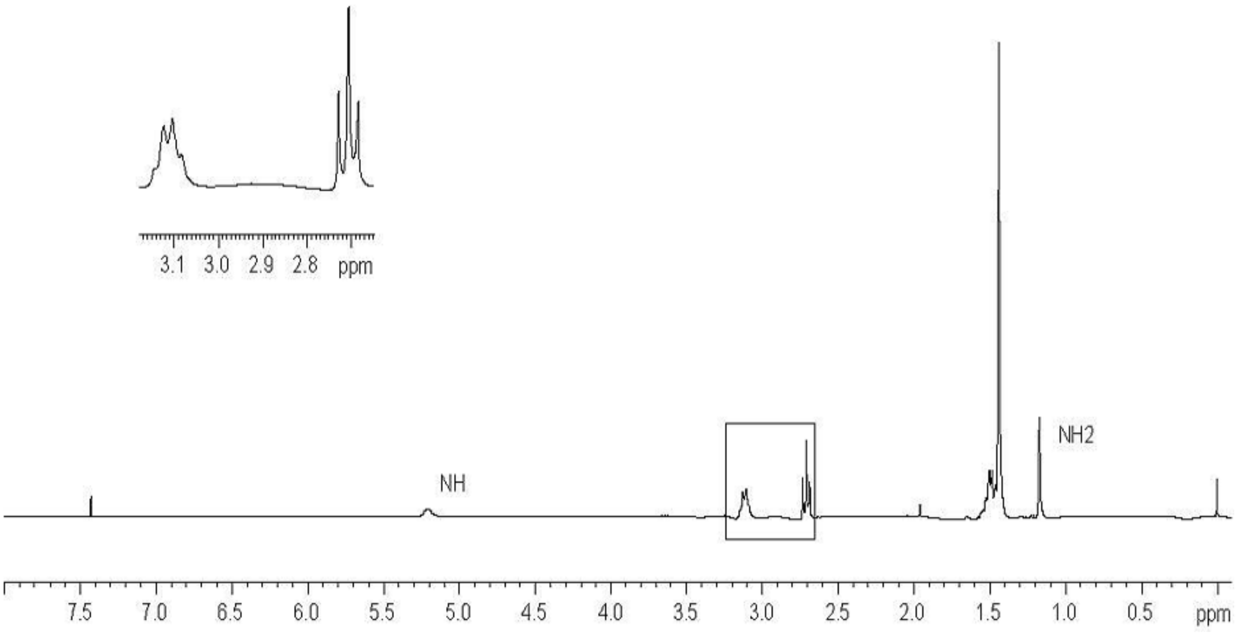


**Figure S7.** $^{1}$H NMR ($CDCl_3$, 300 MHz) spectrum of **6b**.

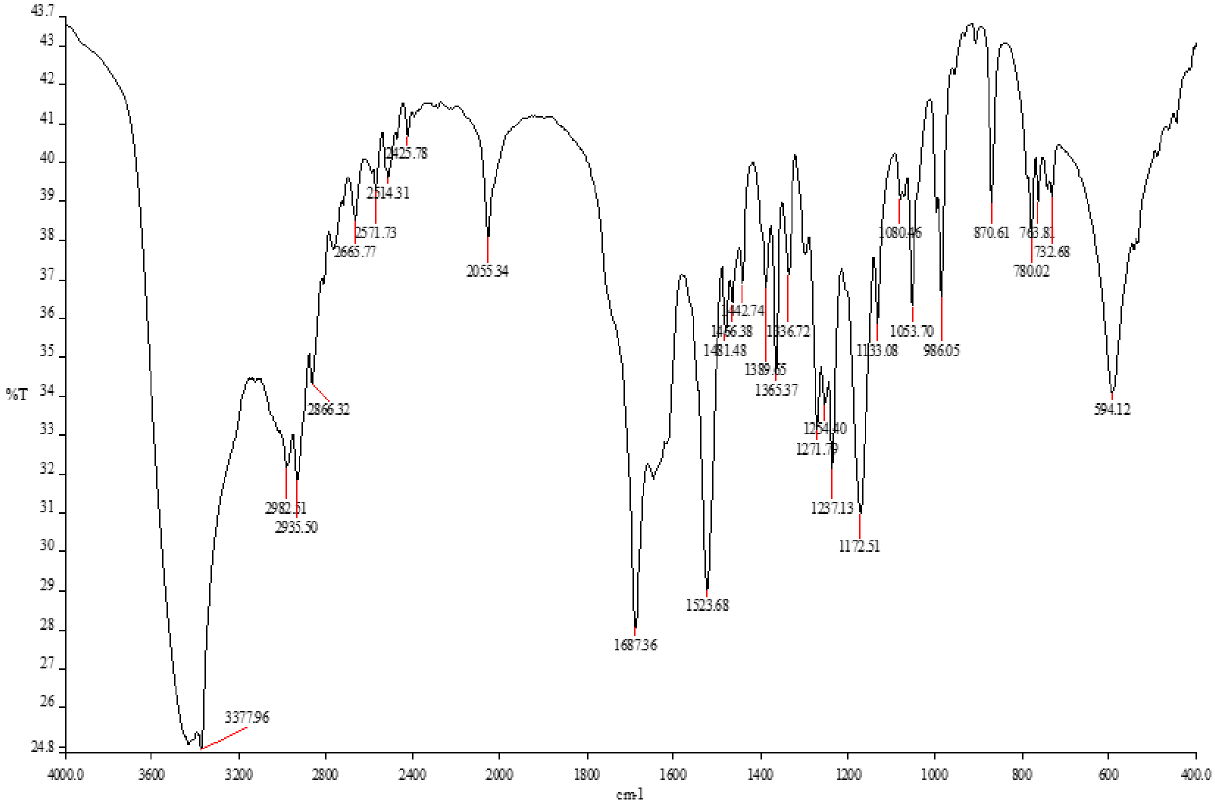


**Figure S8.** FTIR (KBr) spectrum of the hydrochloride salt of **6c**.

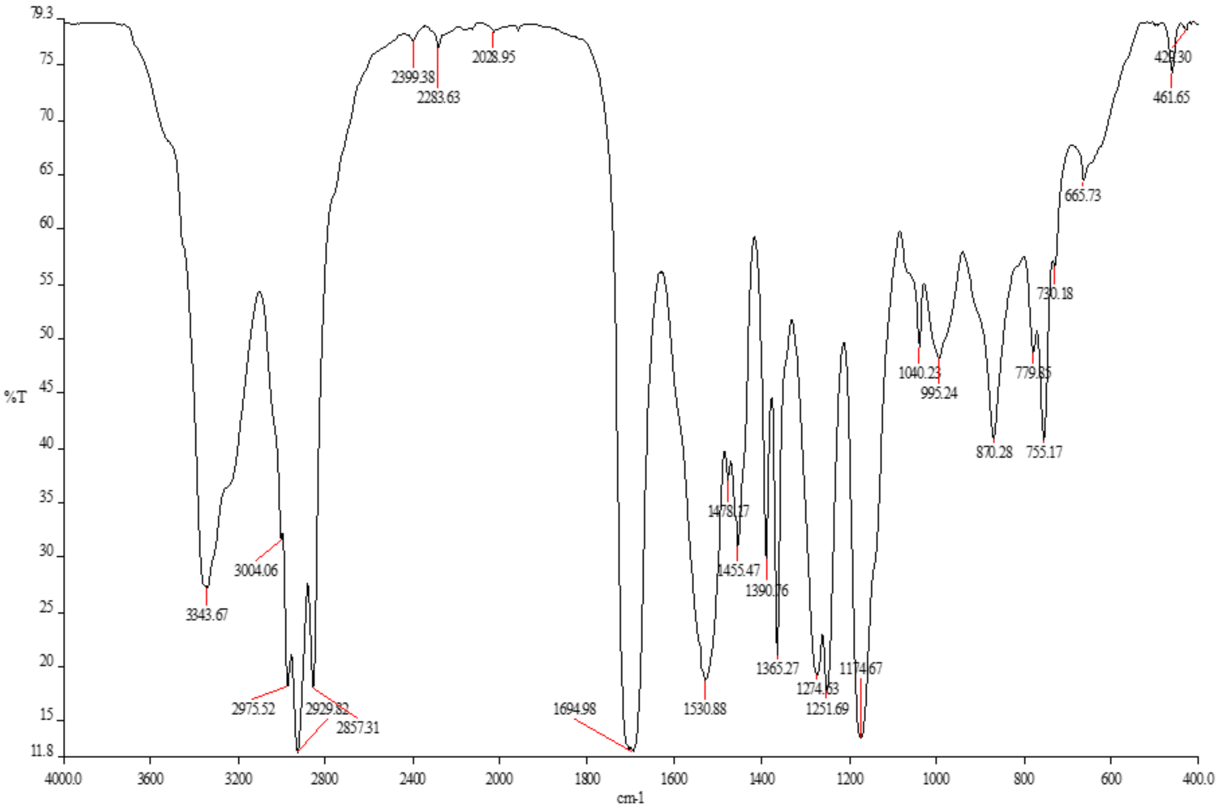


**Figure S9.** FTIR (film) spectrum of **6c**.

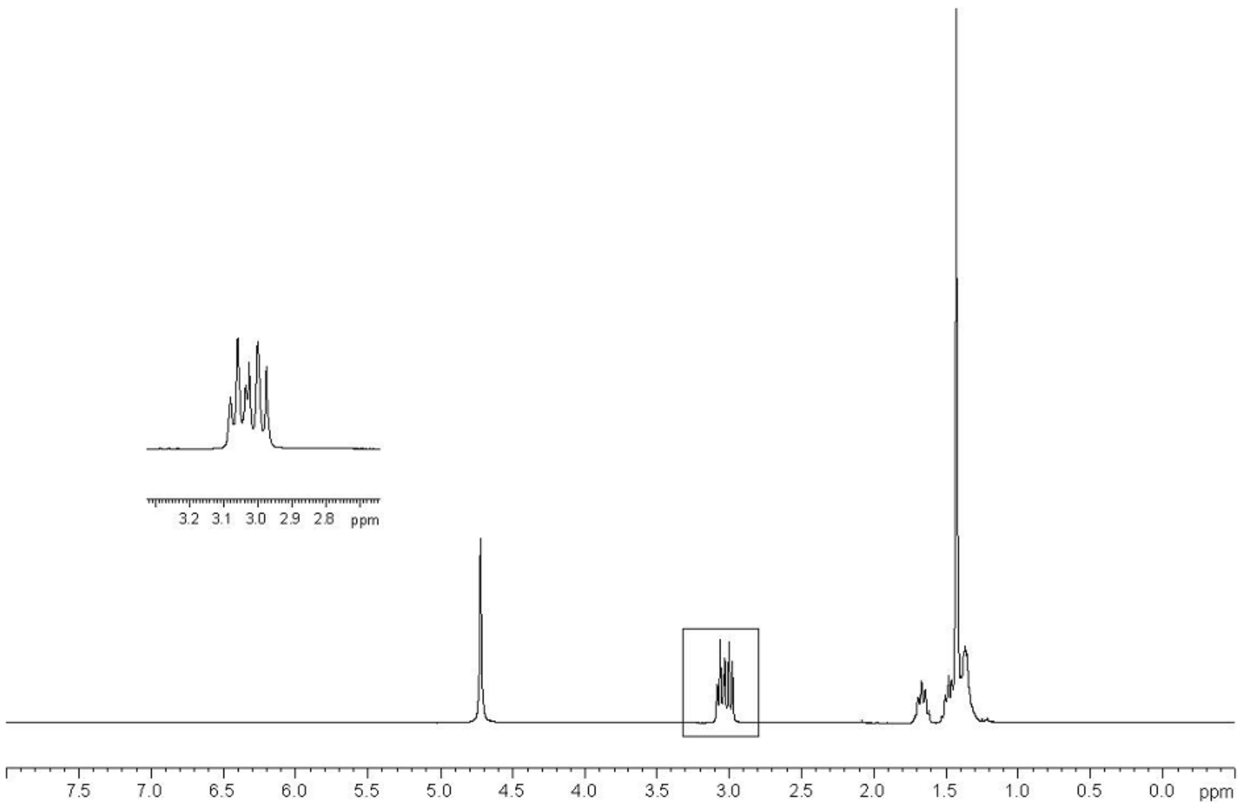


**Figure S10.** $^{1}$H NMR ($D_2O$, 300 MHz) spectrum of the hydrochloride salt of **6c**.

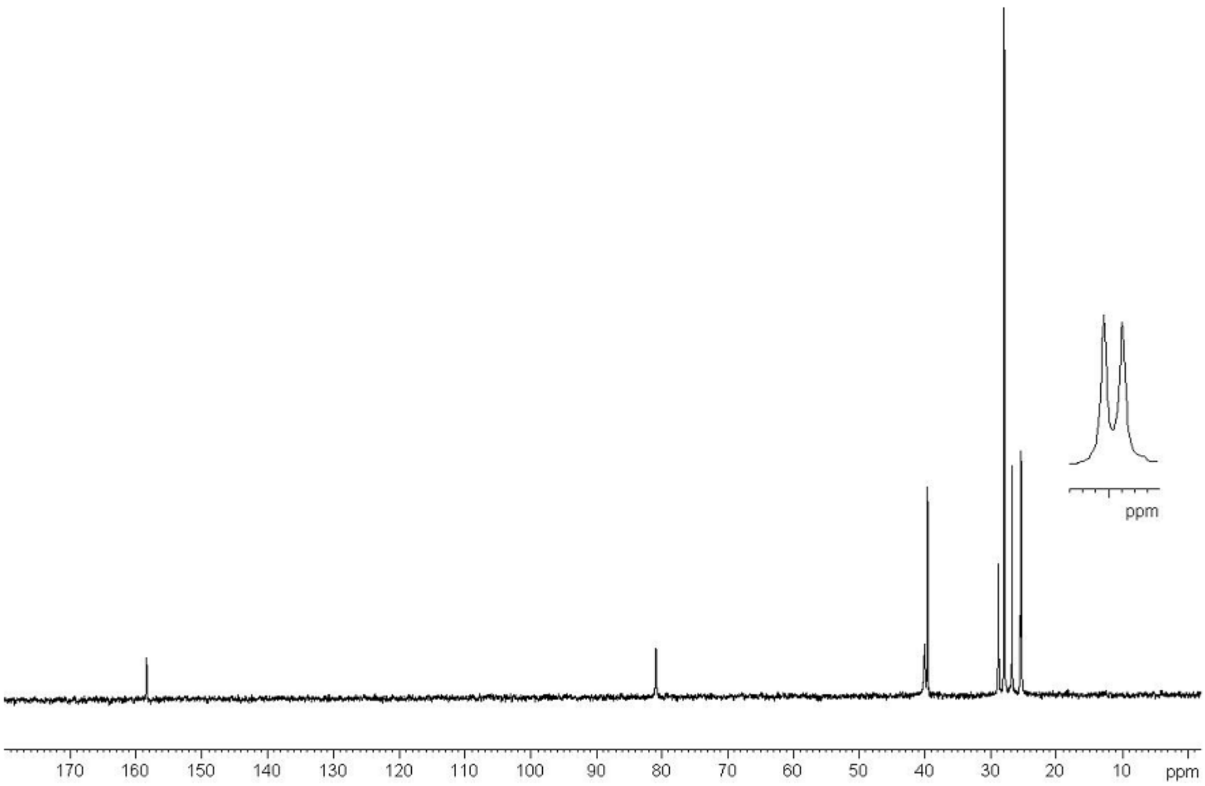


**Figure S11.** $^{13}$C NMR ($D_2O$, 75.5 MHz) spectrum of the hydrochloride salt of **6c**.

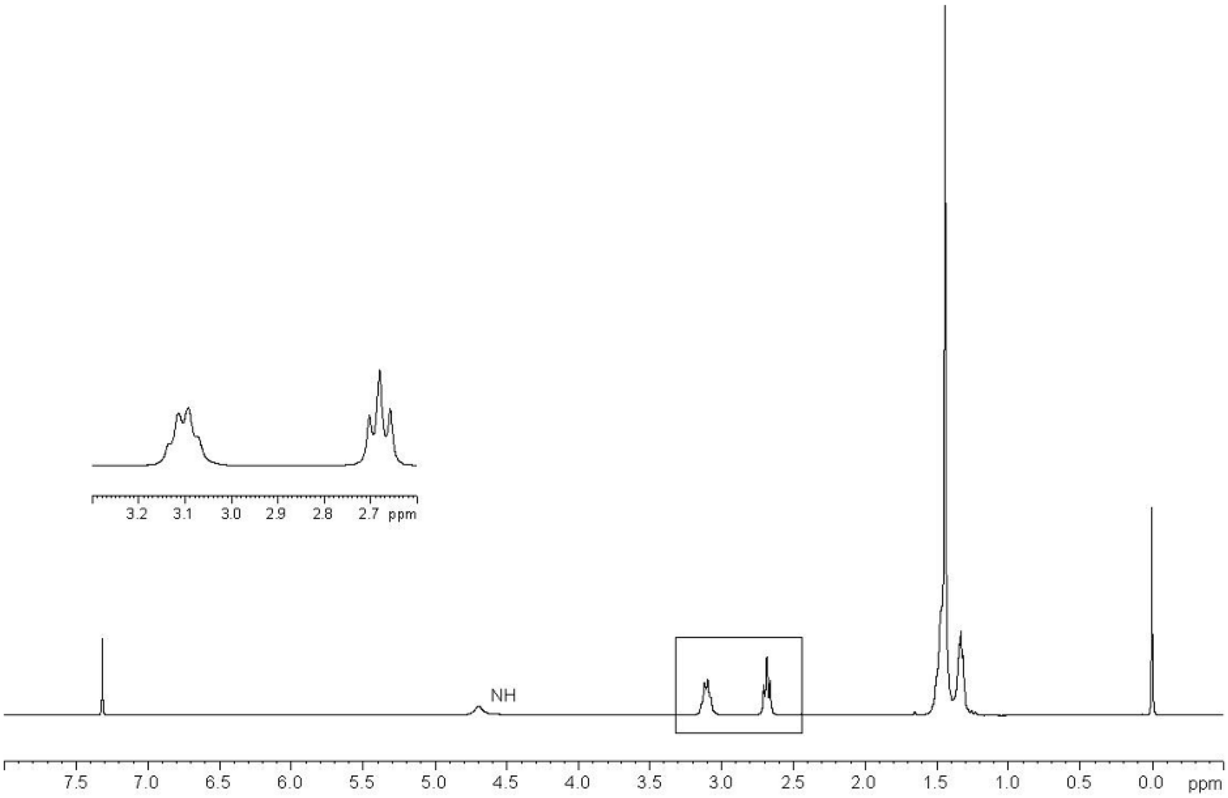


**Figure S12.** $^{1}$H NMR ($CHCl_3$, 300 MHz) spectrum of **6c**.

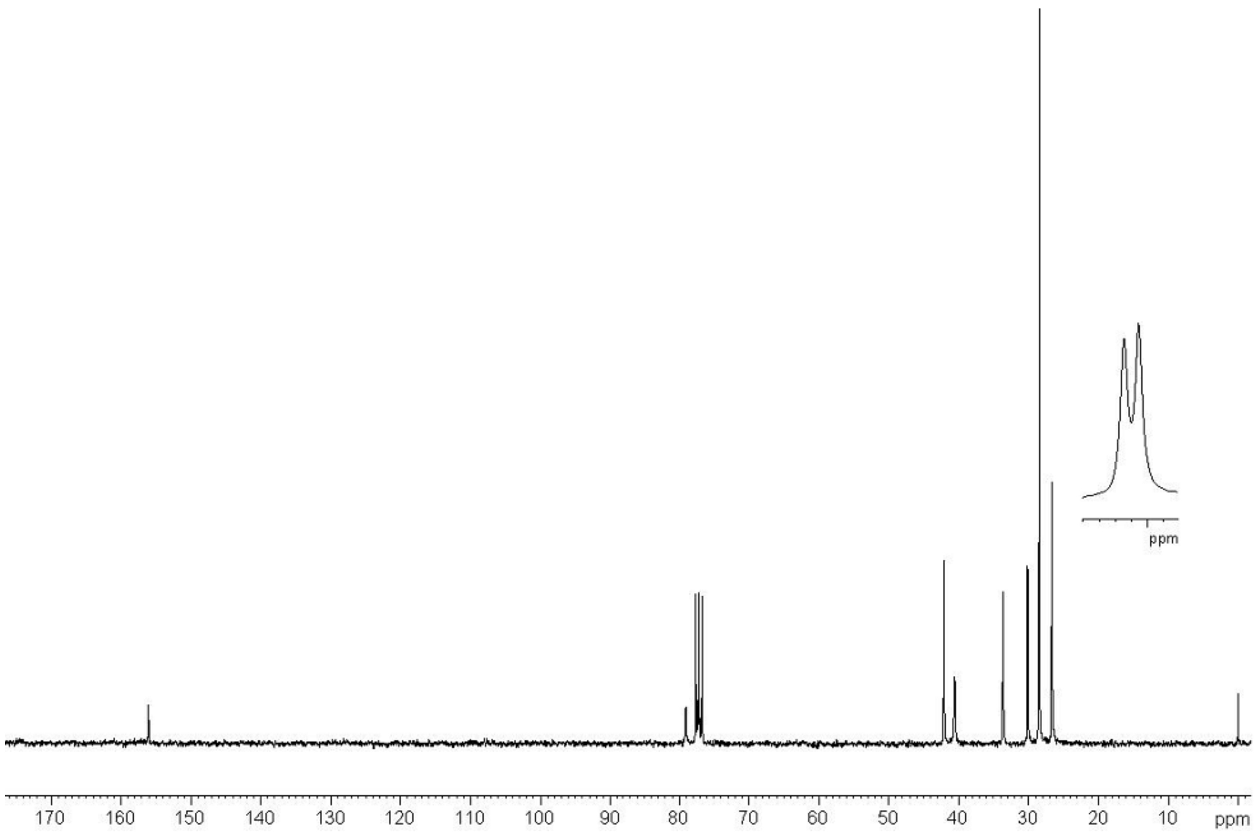


**Figure S13.** $^{13}C$ NMR ($CHCl_3$, 300 MHz) spectrum of **6c**.

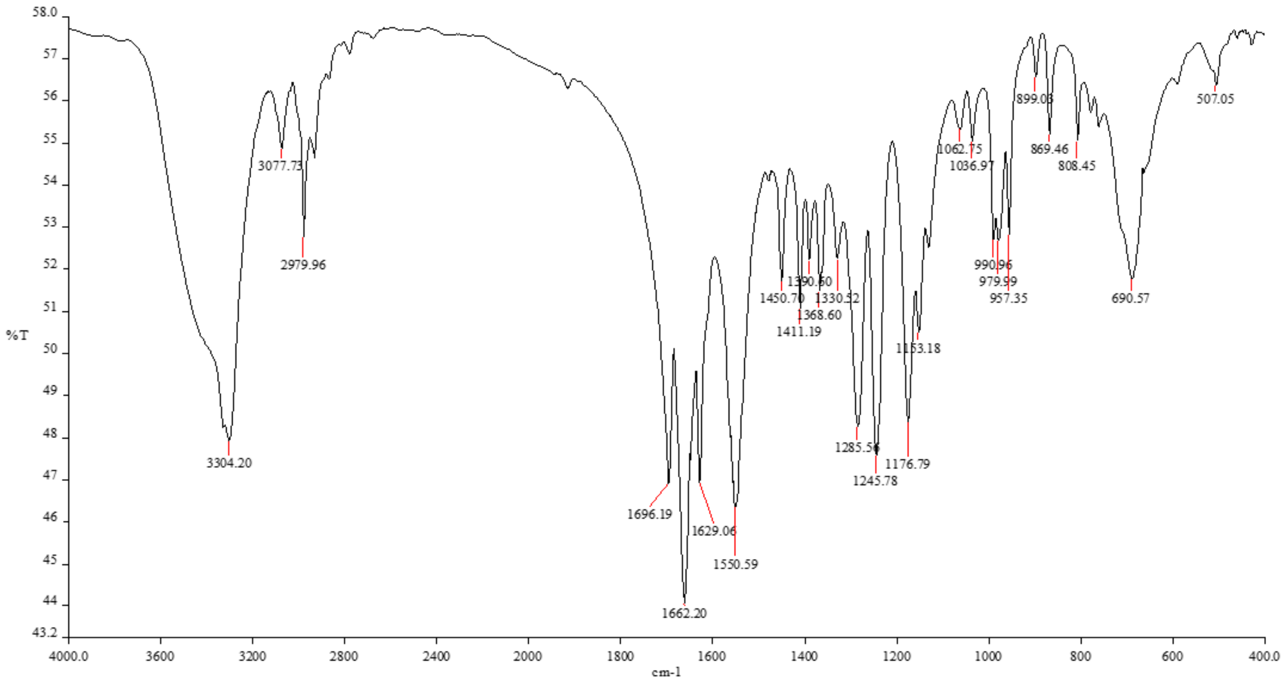


**Figure S14.** FTIR spectrum (KBr) of compound **10a**.

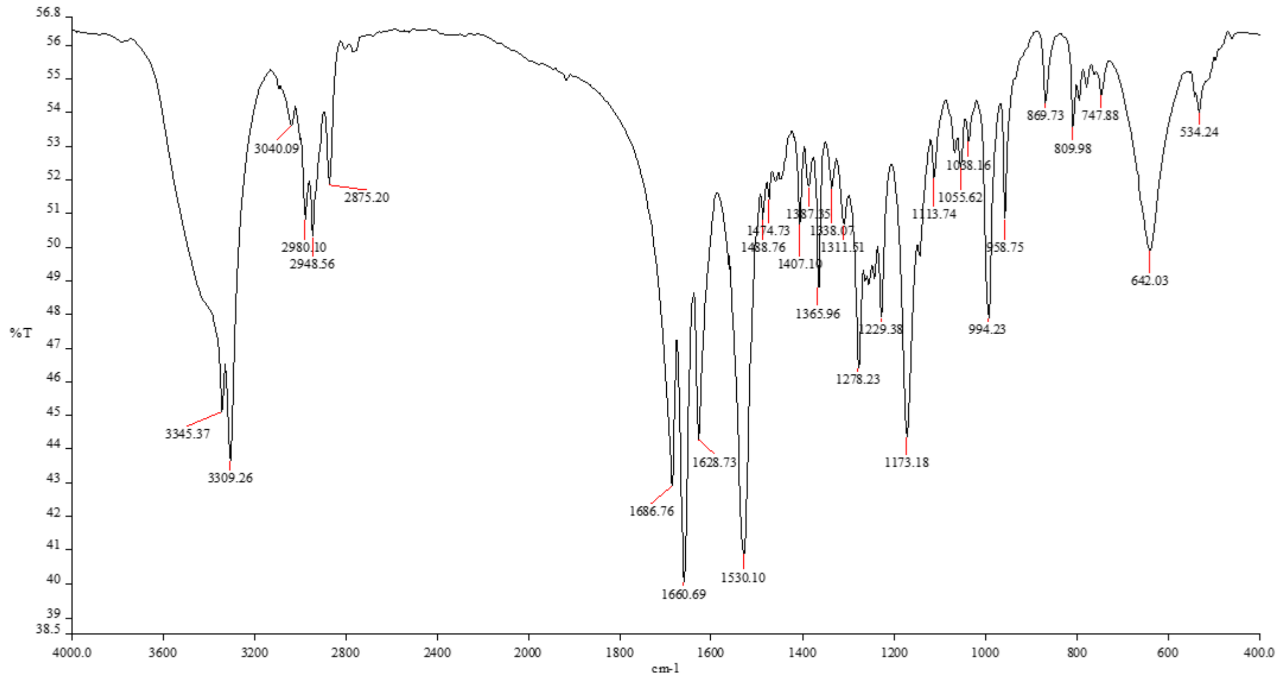


**Figure S15.** FTIR spectrum (KBr) of compound **10b**.

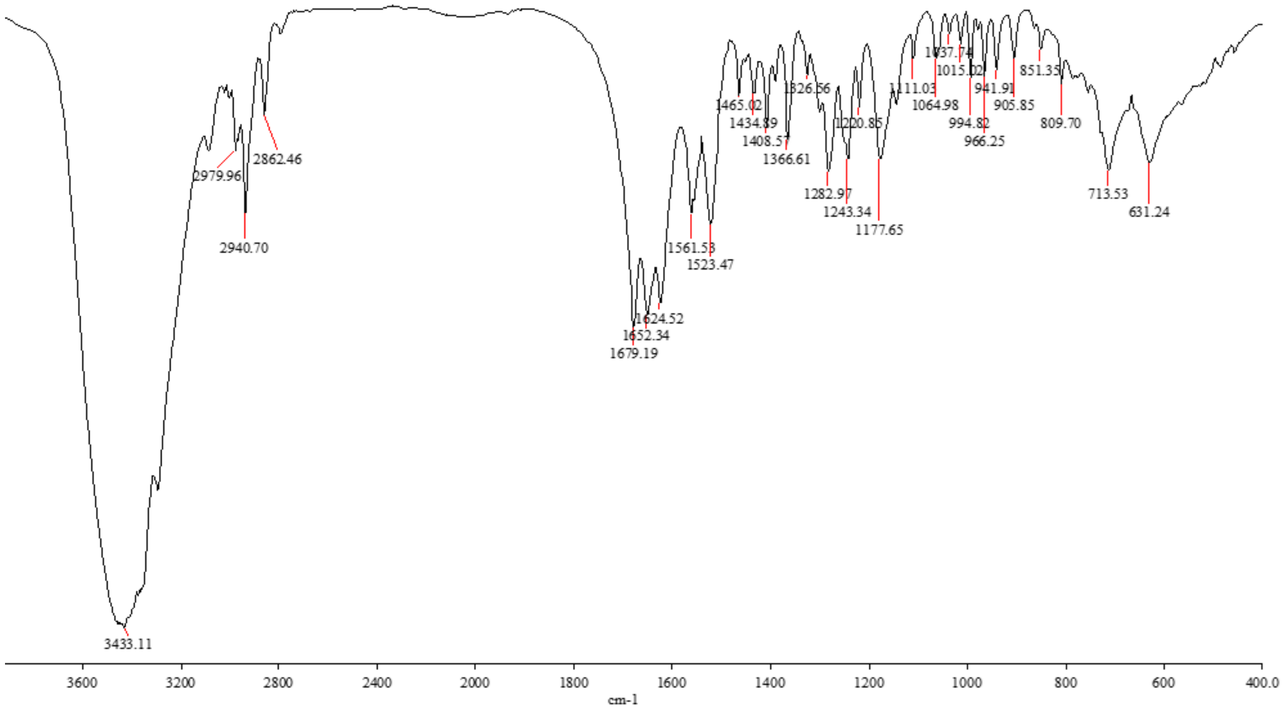


**Figure S16.** FTIR spectrum (KBr) of compound **10c**.

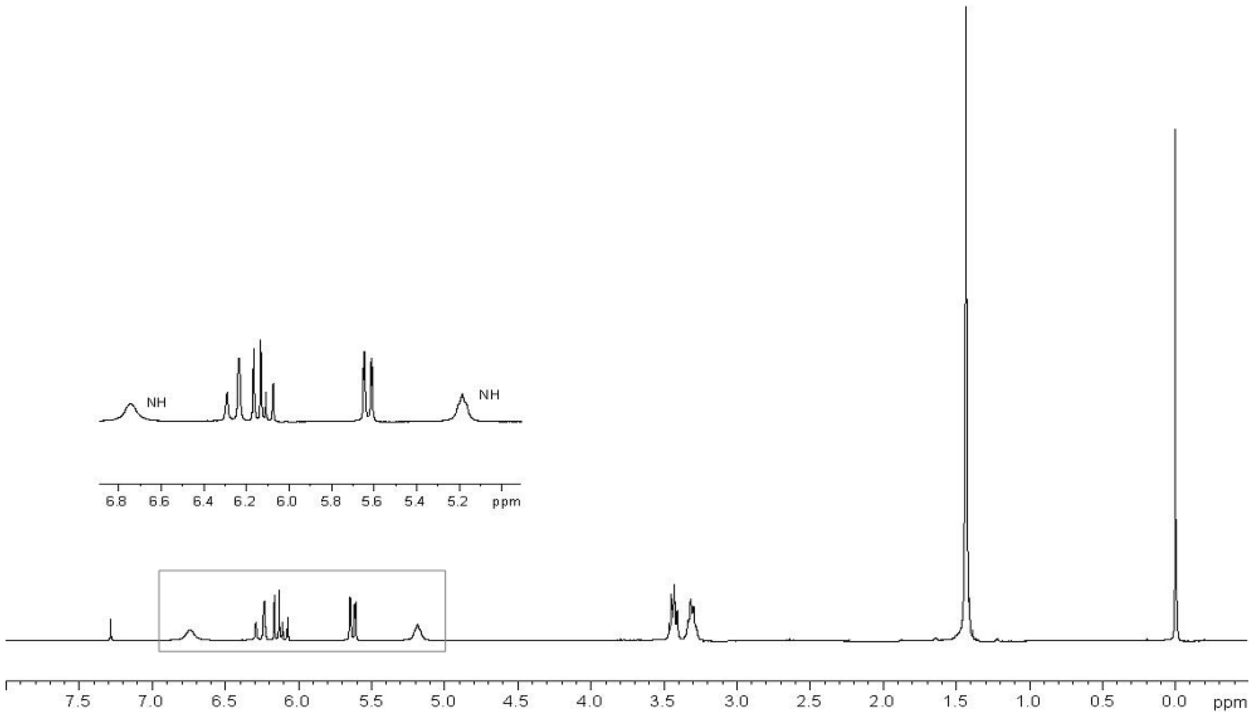


**Figure S17.** $^{1}H$ NMR spectrum ($CHCl_3$, 300 MHz) of compound **10a**.

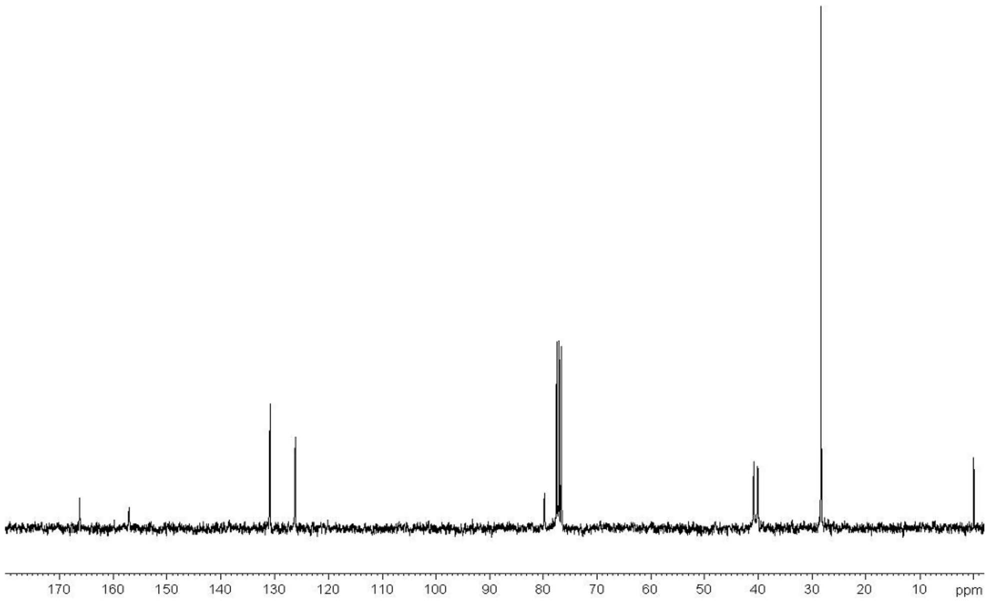


**Figure S18.** $^{13}C$ NMR spectrum ($CHCl_3$, 75.5 MHz) of compound **10a**.

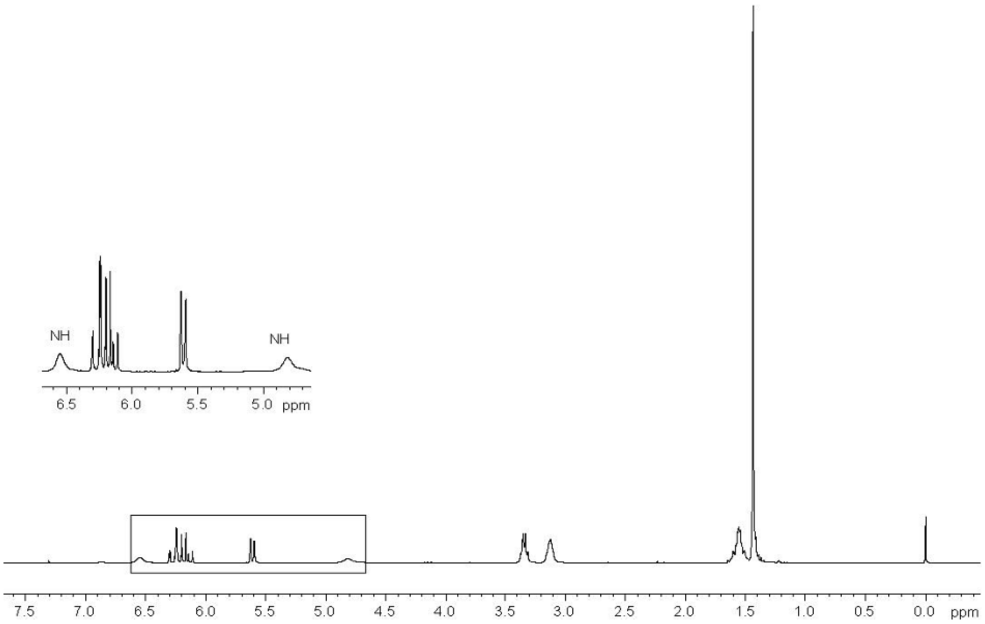


**Figure S19.** $^{1}H$ NMR spectrum ($CHCl_3$, 300 MHz) of compound **10b**.

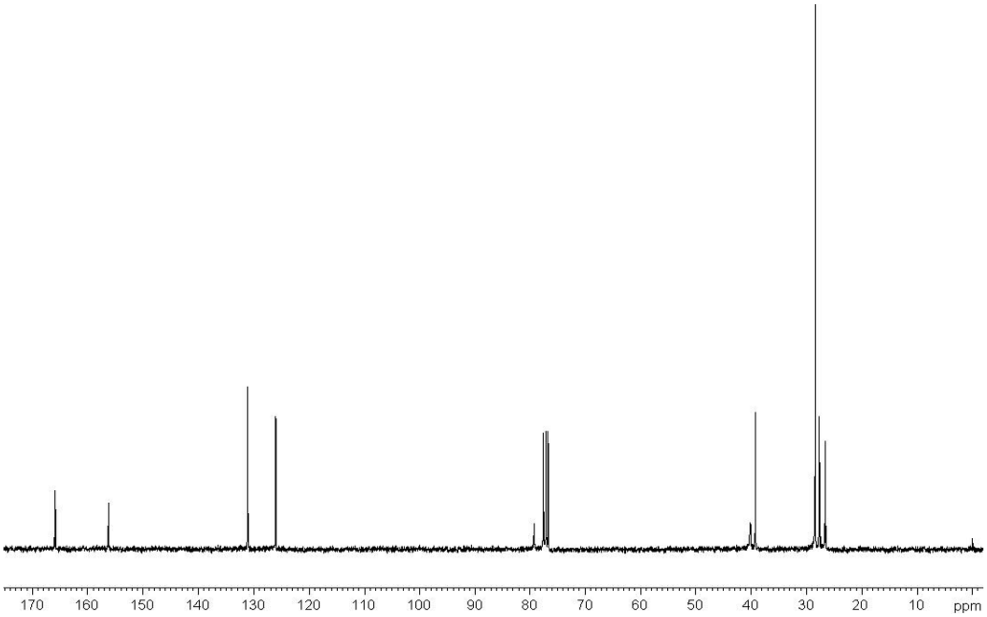


**Figure S20.** $^{13}C$ NMR spectrum ($CHCl_3$, 75.5 MHz) of compound **10b**.

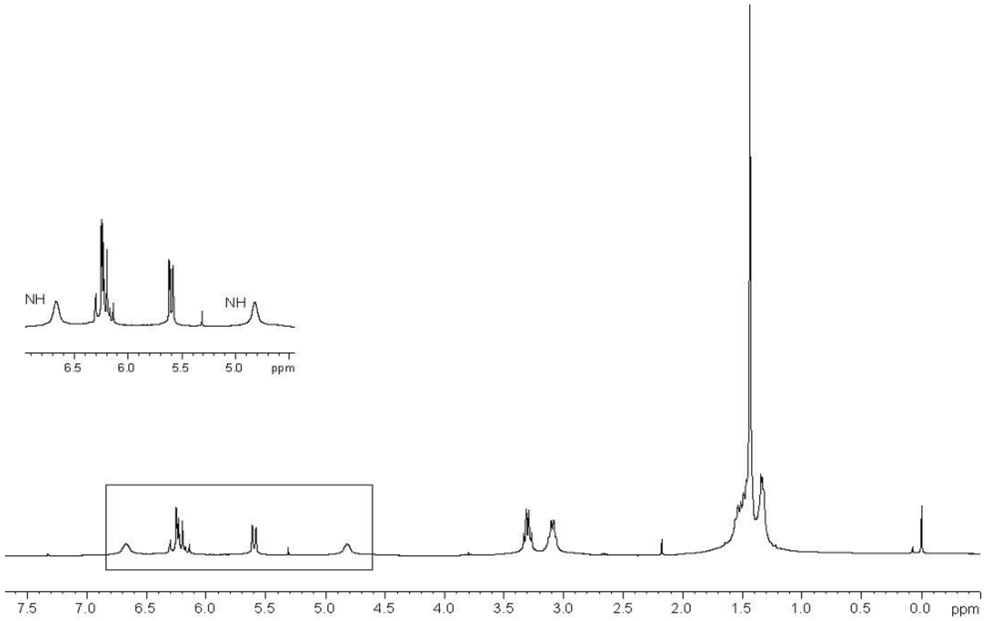


**Figure S21.** $^{1}$H NMR spectrum ($CHCl_3$, 300 MHz) of compound **10c**.

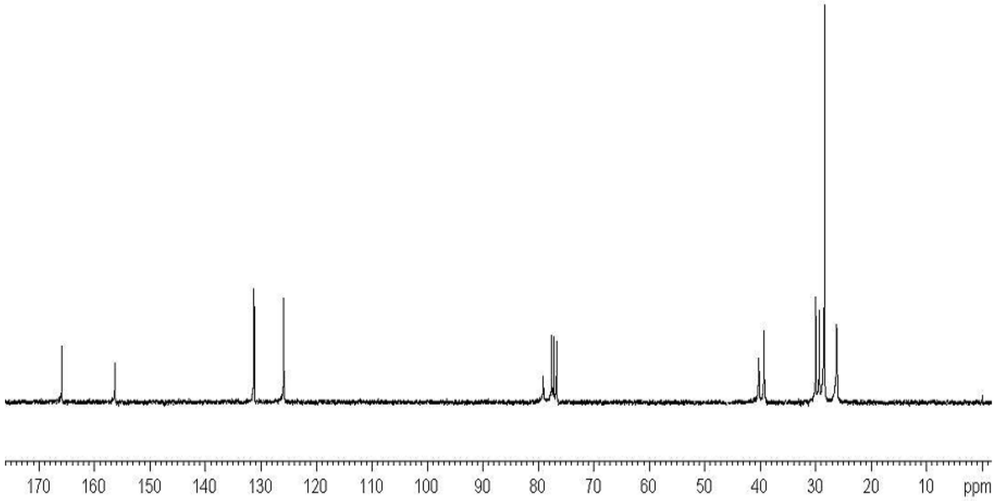


**Figure S22.** $^{13}$C NMR spectrum ($CHCl_3$, 75.5 MHz) of compound **10c**.

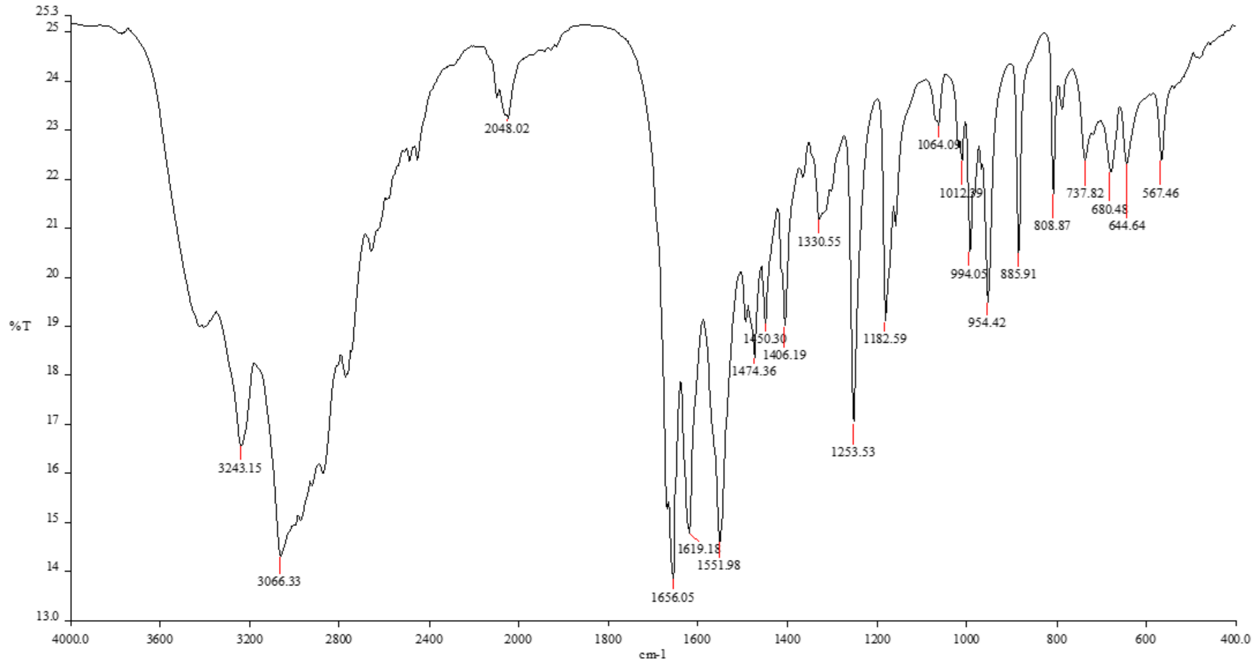


**Figure S23.** FTIR spectrum (KBr) of compound **11a**.

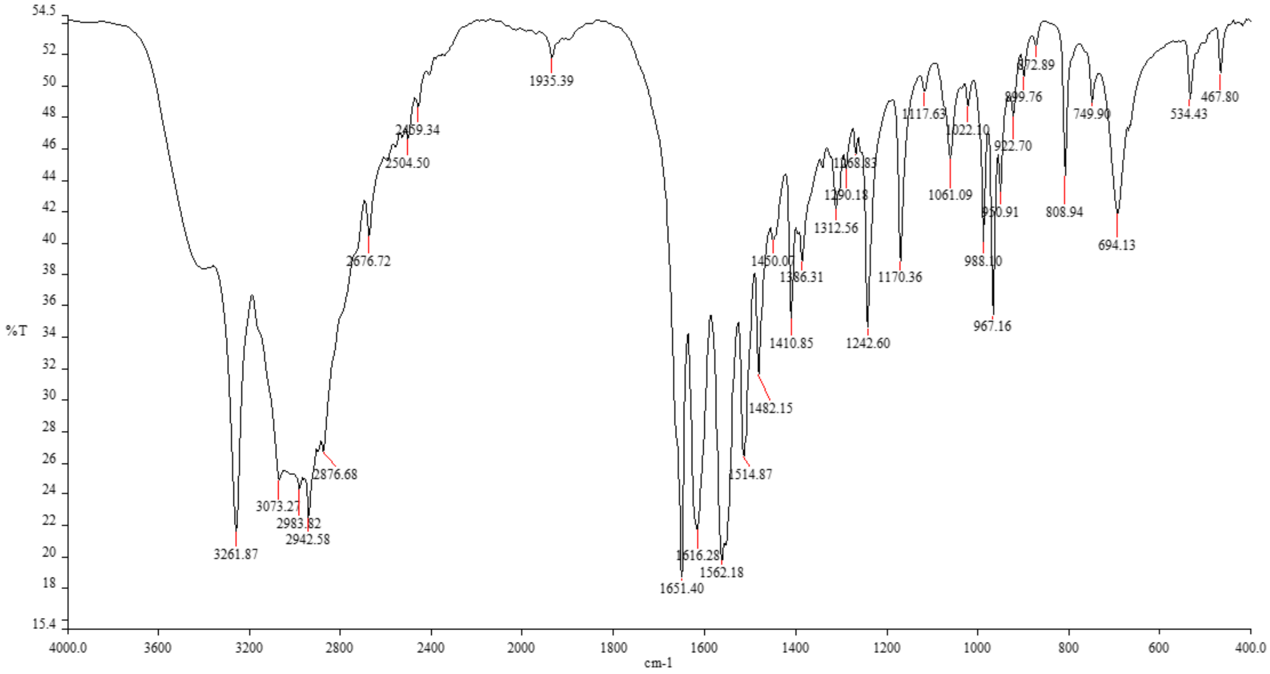


**Figure S24.** FTIR spectrum (KBr) of compound **11b**.

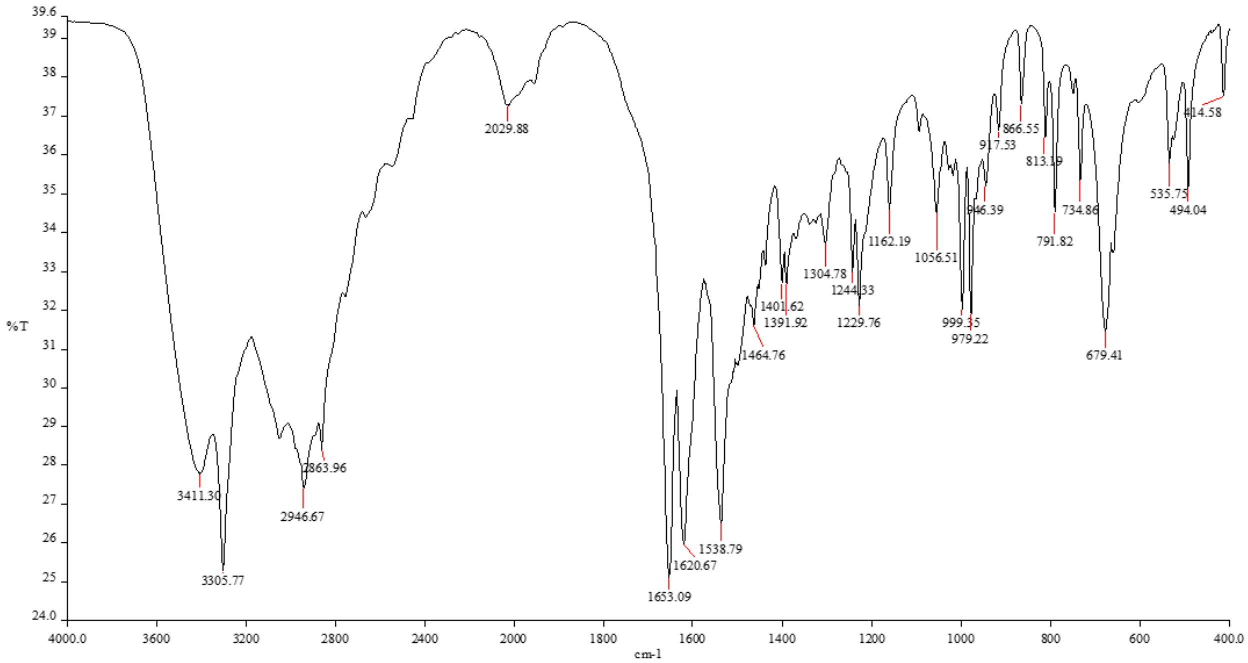


**Figure S25.** FTIR spectrum (KBr) of compound **11c**.

**Table S4.** Solubility of the prepared copolymers and of the DMAA homopolymer prepared for comparison purposes.

| Solvent | CP10a/DMAA | CP10b/DMAA | CP11a/DMAA | CP11b/DMAA | CP11c/DMAA | HP-DMAA |
|---|---|---|---|---|---|---|
| Petrol | - | - | - | - | - | - |
| $Et_2O$ | - | - | - | - | - | - |
| Toluene | Partially soluble * | Partially soluble * | Swells * | Swells * | Swells * | Partially soluble |
| THF | + | + | Swells * | Swells * | Swells * | Soluble * |
| Dioxane | + | + | Swells * | Swells ** | Swells ** | Soluble * |
| Acetone | + | + | Swells * | Swells * | Swells ** | + |
| $CHCl_3$ | + | + | + | + | Soluble * | + |
| DCM | + | + | + | + | + | + |
| MeOH | + | + | + | + | + | + |
| DMF | + | + | + | + | + | + |
| DMSO | + | + | + | + | + | + |
| $H_2O$ | + | + | + | + | + | + |

* Under heating; ** under cooling; petrol = petrol ether boiling at 40–60 °C; DMAA = *N,N′*-dimethylacrylamide; THF = tetrahydrofuran; DCM = dichloromethane; DMF = *N,N*-dimethylformamide; DMSO = dimethyl sulfoxide; HP-DMAA = DMAA homopolymer.

**Table S5.** Main features of commercially available Gel B used in this study.

| Bloom grade (Bloom) | FTIR (cm $^{-1}$) | $NH_2$ (mmol/g) | |
|---|---|---|---|
| 250 | 1645, 1540 * | 0.391± 0.02 ** | 0.219± 0.03 *** |

* C=O amides; ** acid-base titration; *** UV determination; Bloom grade = unit of measurement of the solidity of a gel.

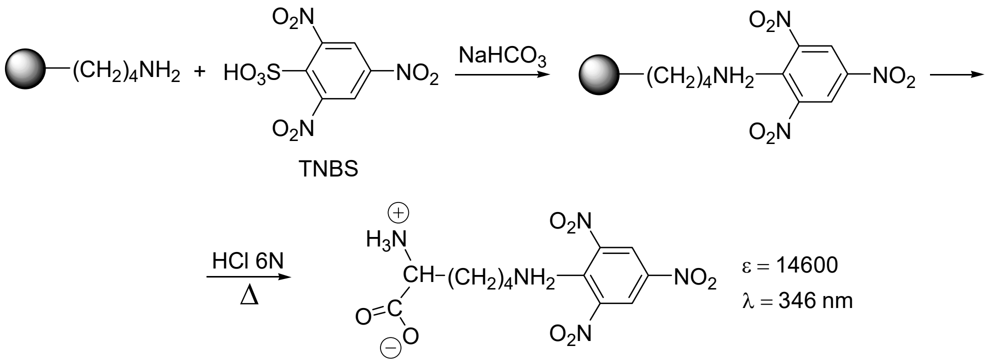


**Scheme S1.** Reaction between gelatine and TNBS leading to the formation of chromophore measurable by UV-Vis analyses.

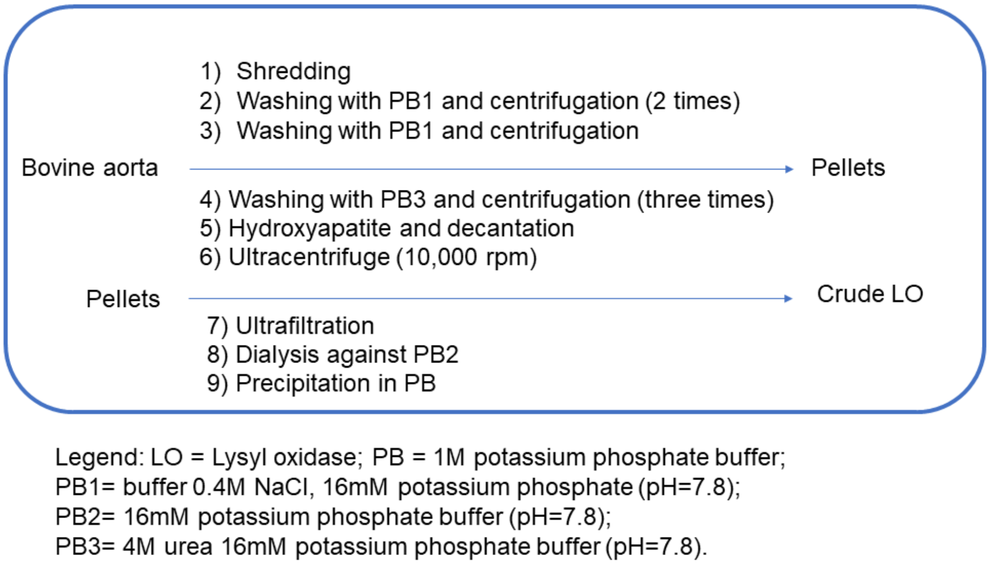


**Chart S1.** Main steps of procedure performed to extract and purify LO from bovine aorta.

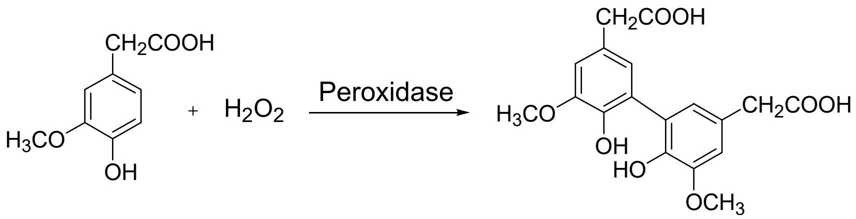


**Scheme S2.** Reaction between homo vanillic acid and hydrogen peroxide in presence of peroxidase leading to the formation of the fluorescent dimer measurable by fluorimetry.

**Table S6.** Oxidation assays of selected copolymers with copper-containing amino oxidases (CAO).

| Entry | Copolymer (mg) | Solvent (μL) | CAO (μL) | CAO stock solution (mg/mL) | Schiff test |
|---|---|---|---|---|---|
| 1 | HP-DMAA 7.1 | $H_2O$ mQ 250 | - | - | - |
| 2 | CP5/DMAA-43.1 * 4.7 | BU6M 500 | LO 150 | 152.5/1.50 BU6M | + |
| 3 | CP5/DMAA-43.1 * 10.2 | BU6M 500 | PAO 400 | 4.8/1.00 BU6M | + |
| 4 | CP5/DMAA-11.3 * 28.3 | BU6M 250 | LO 450 | 159.0/1.08 BU6M | + ** |
| 5 | CP5/DMAA-11.3 * 24.3 | BU6M 250 | PAO 250 | 4.6/0.75 BU6M | - |
| 6 | CP5/DMAA-5.0 * 23.5 | BU6M 250 | LO 450 | 159.0/1.08 BU6M | + ** |
| 7 | CP5/DMAA-5.0 * 23.5 | BU6M 250 | PAO 250 | 4.6/0.75 BU6M | - |
| 8 | TP5/DMAA/AA=1/8/1 10.0 | BU6M 250 | LO 250 | 152.5/1.50 BU6M | - |
| 9 | TP5/DMAA/AA=1/8/1 9.2 | BU6M 250 | PAO 250 | 4.8/0.75 BU6M | - |
| 10 | CP11a/DMAA-10.0 * 17.1 | BU6M 250 | LO 450 | 152.5/1.50 BU6M | - |
| 11 | CP11a/DMAA-10.0 * 8.8 | BU6M 250 | PAO 200 | 4.8/1.25 BU6M | - |
| 12 | CP11b/DMAA-10.0 * 15.3 | BU6M 250 | LO 600 | 46.0/0.60 BU6M | + |
| 13 | CP11b/DMAA-10.0 * 14.7 | BU6M 250 | PAO 200 | 1.2/0.20 BU6M | + |
| 14 | CP11c/DMAA-9.9 * 13.3 | BU6M 250 | LO 450 | 152.5/1.50 BU6M | +/- |
| 15 | CP11c/DMAA-9.9 * 16.8 | BU6M 250 | PAO 200 | 4.8/1.25 BU6M | + |

* The code number indicates the percentage of monomers in the reaction feed; ** heating; CAO = copper-containing amino oxidases; **5** = 4-aminobutylstyrene hydrochloride; DMAA = *N*,*N*-dimethylacrylamide; BU6M= borate buffer 0.05 M, Urea 6M; LO = lysyl oxidase; PAO = plasma amine oxidase; **11a** = *N*-acryloyl-1,2-diaminoethane hydrochloride; **11b** = *N*-acryloyl-1,4-diaminobutane hydrochloride; **11c** = *N*-acryloyl-1,6-diaminohexane hydrochloride.

**Table S7.** Size, PDI and ζ-p of CP5/DMAA, CP11b/DMAA, CP11c/DMAA and CPMA/DMAA.

| Entry | Z AVE (nm) | PDI | ζ-p (mV) |
|---|---|---|---|
| CP5/DMAA | 334.0 ± 27.0 | 1.012 | +57.6 ± 1.7 |
| CP11b/DMAA | 2590 ± 56.7 | 0.281 | +6.5 ± 3.7 |
| CP11c/DMAA | 372.8 ± 196.8 | 0.326 | +24.8 ± 5.1 |
| CPMA/DMAA | 112.2 ± 82.8 | 0.590 | +18.3 ± 9.9 |

**Table S8.** Crosslinking of gelatine with copolymers.

| Exp | Gel B (mg) | Solvent (mL) | Copolymer (mg) | Wt % | Solvent mL | CAO mL | Gel B Crosslinked (mg, %) |
|---|---|---|---|---|---|---|---|
| 1 | 500.1 | BU6M 5.0 | CP5/DMAA-43.1 50.5 | 10.1 | - | LO§ 2.0 | M21 76.6, 13.9 |
| 2 | 503.1 | BU6M 4.0 | CP11b/DMAA-10.0 51.5 | 10.2 | BU6M 1.0 | LO† 5.0 | M33 1.9, 0.38 |
| 3 | 502.7 | BU6M 4.0 | CP11c/DMAA-10.0 52.4 | 10.4 | BU6M 1.0 | PAO‡ 0.600 | M31 37.3, 7.4 |
| 4 | 500.8 | PB 4.0 | CPMA/DMAA-20.0 51 | 10.2 | PB 2.0 | - | M23 498.7, 99.5 |
| 5 | 500.6 | PB 4.0 | CPMA/DMAA-20.0 25.4 | 5.1 | PB 1.0 | - | M26 345.5, 69.0 |
| 6 | 500.2* | PB 6.5 | CPMA/DMAA-20.0 25.5 | 5.1 | $H_2O$ mQ 1.0 | - | M32 310.7, 62.1 |
| 7 | 650.1 | BU6M 5.2 | CPMA/DMAA-20.0 65.0 | 10.0 | BU6M 1.5 | LO♯ 5.2 | M34 399.8, 57.2 |

**5** = 4-aminobutylstyrene hydrochloride; DMAA = *N*,*N*-dimethylacrylamide; MA = methacrolein; **11c** = *N*-acryloyl-1,6-diaminohexane hydrochloride; **11b** = *N*-acryloyl-1,4-diaminobutane hydrochloride; LO = lysyl oxidase; PAO = plasma amine oxidase; BU6M = 0.05 M borate buffer, 6M Urea (pH = 8.1 - 8.3); PB (phosphate, pH = 7.8); * gelatine was added to a hyaluronic acid (HA) solution obtained by dissolving 10.2 mg of HA in 5 mL of phosphate buffer. § 0.5029g /5.0 mL BU6M; ‡ 0.0037g/0.6 mL BU6M; † 0.4092/5.5 mL BU6M; ♯ 0.5254/5.2 mL BU6M.

**Table S9.** UV and NaOH titration of pristine Gel B and of some selected crosslinked derivatives.

| Sample (mg) | CL agent (% *) | $NH_2$/g (UV) (mmol) | $NH_2$/g (NaOH) (mmol) | Crosslinking (%) |
|---|---|---|---|---|
| Gel B | | | | |
| 11.2 | - | 0.219±0.02 | - | - |
| 251.3 | - | - | 0.391±0.03 | - |
| M21 | CP5/DMAA-43.1 | | | |
| 11.4 | 10.1 | 0.065±0.001 | - | 70.5 |
| M31 | CP11c/DMAA-10.0 | | | |
| 11.9 | 10.4 | 0.208±0.04 | - | 5.0 |
| 220.1 | 10.4 | - | 0.372±0.03 | 4.9 |
| M23 | CPMA/DMAA-20.0 | | | |
| 11.4 | 10.2 | 0.207±0.03 | - | 5.5 |
| 222.9 | 10.2 | - | 0.157±0.02 | 59.8 |
| M26 | CPMA/DMAA-20.0 | | | |
| 11.6 | 5.1 | 0.225±0.02 | - | ≈ 0 |
| 221.9 | 5.1 | - | 0.190±0.01 | 51.4 |
| M32 | CPMA/DMAA-20.0 | | | |
| 12.9 | 5.1 | 0.174±0.01 | - | 20.5 |
| 220.5 | 5.1 | - | 0.164±0.01 | 58.1 |
| M34 | CPMA/DMAA-20.0 | | | |
| 11.5 | 10.0 | 0.118±0.015 | - | 46.1 |

CL = Crosslinking; * % wt/wt with Gel B.

**Table S10.** Experimental data of the synthesis of compounds **6a, b**.

| Entry (g, mmol, mL) | DCM (mL) | $Boc_2O$ (g, mmol) | Time (h) | Side product (g, mmol, %) | Product (g, mmol, %) | Physical state |
|---|---|---|---|---|---|---|
| 1,2-ethylenediamine 5.39, 89.68, 6.0 | 50 * 25 ** | 4.90, 22.45 | 21 | **7a** 0.6067, 2.33, 10.4 | **6a** 2.64, 16.45, 73.3 | Light-yellow oil |
| 1,4-diaminobutane 6.14, 69.65, 7.0 | 70 * 30 ** | 3.80, 17.39 | 21 | **7b** 0.3896, 1.35, 7.8 | **6b** 3.13, 16.64, 95.7 | Yellow oil |

$Boc_2O$ = *t*-butyl-di-carbonate; * for dissolving diamines; ** for dissolving $Boc_2O$.

**Table S11.** Experimental data of the synthesis of compounds **10a-c**.

| Entry (g, mmol) | Solvent (mL) | Acryloyl chloride (g, mmol, mL) | TEA (g, mmol, mL) | Time (min) | Product (g, mmol, %) | Physical state |
|---|---|---|---|---|---|---|
| **6a** 2.17, 13.57 | 32.5 * 16.5 ** | 1.43, 16.29, 1.35 | 1.37, 13.57, 1.9 | 90 ***/135 ° | **10a** 2.51, 11.71, 86.3 | Solid |
| **6b** 3.01, 16.01 | 38.0 * 19.5 ** | 1.7, 19.21, 1.6 | 1.6, 16.01, 2.2 | 45 ***/120 ° | **10b** 3.26, 13.45, 84.1 | Solid |
| **6c** 3.04, 14.05 | 34.0 * 17.0 ** | 1.53, 16.86, 1.4 | 1.42, 14.05, 1.95 | 90 ***/overnight ° | **10c** 3.03, 11.21, 79.8 | Solid |

TEA = triethylamine; * for dissolving acryloyl chloride; ** for dissolving compounds **6a-c**; *** for the dropwise adding of TEA and **6a-c** solution; ° reaction time.

**Table S12.** Experimental data of the synthesis of monomers **11a-c**.

| Entry (g, mmol) | EtOAc (mL) | HCl 3N (mL) | HCl 37% (mL) | Time (min) | Product (g, mmol, %) | Physical state |
|---|---|---|---|---|---|---|
| **10a** 2.90, 15.50 | --- | 7.5 | --- | 80 ° | **11a** 1.77, 11.80, 76.1 | Solid |
| **10b** 1.60, 6.61 | 20 | --- | Needed to pH = 1 | N.R. ° | **11b** 0.97, 5.50, 83.2 | Solid * |
| **10c** 2.98, 11.02 | --- | 12 | --- | 60 ° | **11c** 2.25, 10.87, 98.6 | Solid |

° Reaction time; * for characterization the dark solid was recrystallized by acetonitrile ($CH_3CN$).

**Table S13.** Copolymerization reactions of **10a**, **10b** and **11c** with DMAA a 60 °C.

| Entry | mg (mmol) | DMAA (g, mmol) | $M_m$ | AIBN (mg, %) | MeOH (mL) | Time (min) | Copolymer | (g, %) |
|---|---|---|---|---|---|---|---|---|
| **10a** | 201.1 (0.94) | 0.84, 8.47 | 0.0997 | 10.4, 1.00 | 7.0 | 290 | CP10a/DMAA | 0.658, 63.2 |
| **10a** | 200.0 (0.93) | 0.88, 8.40 | 0.0997 | 10.5, 0.97 | 7.0 | 210 | CP10a/DMAA | 0.749, 69.4 |
| **10b** | 200.3 (0.83) | 0.74, 7.47 | 0.0997 | 9.68, 1.03 | 6.5 | 270 | CP10b/DMAA | 0.435, 46.3 |
| **10b** | 400.6 (1.65) | 1.47, 14.85 | 0.1000 | 18.7, 1.00 | 13.0 | 270 | CP10b/DMAA | 1.458, 79.8 |
| **11c** | 100.9 (0.49) | 0.44, 4.44 | 0.0994 | 5.46, 1.01 | 3.0 | 150 | CP11c/DMAA | 0.478, 94.7 |
| **11c** | 201.0 (0.97) | 0.88, 8.75 | 0.0998 | 11.0, 1.02 | 4.0 | 210 | CP11c/DMAA | 0.819, 76.1 |

DMAA= *N, N′*-di-methylacrylamide; $M_m$= molar fractions of the monomers into the feed; AIBN = 2,2'-azo-*bis*-(2-methylpropane nitrile).

**Table S14.** Boc deprotection of copolymers CP10a/DMAA e CP10b/DMAA.

| Copolymer | g | HCl 3N* (mL) | Time(h) | Product | g, % |
|---|---|---|---|---|---|
| CP10a/DMAA | 0.337 | 3.3 | 4 | CP11a/DMAA | 0.277, 82.2 |
| CP10a/DMAA | 0.406 | 4.0 | 4 | CP11a/DMAA | 0.261, 64.3 |
| CP10b/DMAA | 0.267 | 2.0 | 4 | CP11b/DMAA | 0.180, 67.4 |
| CP10b/DMAA | 0.396 | 4.0 | 4 | CP11b/DMAA | 0.376, 94.9 |

* in EtOAc; DMAA = *N, N′*-di-methylacrylamide.
